# Characterization and modeling of the borate transporter BOR3 suggest a mode of directional transport

**DOI:** 10.1093/plphys/kiag463

**Published:** 2026-07-30

**Authors:** Naoyuki Sotta, Shigeki Takada, Kyoko Miwa, Junpei Takano, Yukari Kawata, Verônica A Grieneisen, Toru Fujiwara, Athanasius F M Marée

**Affiliations:** Graduate School of Agriculture, Osaka Metropolitan University, 1-1, Gakuen-cho, Naka-ku, Sakai-shi, Osaka 599-8531, Japan; School of Biosciences, Cardiff University, Sir Martin Evans Building, Museum Avenue, Cardiff CF10 3AX, United Kingdom; Graduate School of Agricultural and Life Sciences, The University of Tokyo, 1-1-1, Yayoi, Bunkyo-ku, Tokyo 113-8657, Japan; Graduate School of Agriculture, Hokkaido University, North-9, West-9, Kita-ku, Sapporo, Hokkaido 060-8589, Japan; Graduate School of Environmental Science, Hokkaido University, North-10, West-5, Kita-ku, Sapporo, Hokkaido 060-0810, Japan; Graduate School of Agriculture, Osaka Metropolitan University, 1-1, Gakuen-cho, Naka-ku, Sakai-shi, Osaka 599-8531, Japan; Graduate School of Agriculture, Hokkaido University, North-9, West-9, Kita-ku, Sapporo, Hokkaido 060-8589, Japan; Graduate School of Agricultural and Life Sciences, The University of Tokyo, 1-1-1, Yayoi, Bunkyo-ku, Tokyo 113-8657, Japan; School of Biosciences, Cardiff University, Sir Martin Evans Building, Museum Avenue, Cardiff CF10 3AX, United Kingdom; Graduate School of Agricultural and Life Sciences, The University of Tokyo, 1-1-1, Yayoi, Bunkyo-ku, Tokyo 113-8657, Japan; School of Biosciences, Cardiff University, Sir Martin Evans Building, Museum Avenue, Cardiff CF10 3AX, United Kingdom

## Abstract

In *Arabidopsis thaliana*, BOR1 and BOR2 are well-characterized borate exporters. The role of the paralog BOR3, however, has remained undetermined. We found that the *bor1 bor2 bor3* triple mutant exhibits significantly poorer root elongation than the double *bor1 bor2* mutant, suggesting that BOR3 contributes to the uptake of boron from the soil to the inner tissues in the absence of BOR1 and BOR2. In contrast to BOR1 and BOR2, BOR3 localization in the plasma membrane of the root cells was not noticeably polar, as would be expected given its ability to promote directional boron transport under the triple mutant background. To understand this apparent dichotomy between function and localization, we modeled the effect of BOR3 polarity on boron flow. It revealed that a synergy of diffusive processes can lead to directional transport within a plant tissue. The counterintuitive mechanism, here termed *diffusive alliance transport* (DAT) consists of perfectly apolar (or even outward-facing) exporters working in conjunction with polarly localized diffusion facilitators to enable the buildup of higher-than-medium concentrations as well as inward-directional flux within the tissue. For this mechanism to be operational, the existence of apoplastic compartments between cells is essential.

## Teaser

A boron exporter helps roots absorb boron, although it is evenly distributed rather than on one side of the cell, showing exporters with one-sided channels can drive directional flow against gradients.

## Introduction

Plant roots take up essential elements from the soil. When these are in low quantities in the external medium, it is paramount that the root system actively establishes uptake through nondiffusive processes. Boron, an essential element for plant growth and patterning (Warington 1923), is taken up by the root through a network of well-characterized channels and transporters. NOD26-Like intrinsic Protein5;1 (NIP5;1) is a channel that allows boric acid to enter the root by increasing the diffusive permeability of the cell's plasma membrane (PM) at its location (Takano et al. 2006). NIP5;1 is characterized to be localized to the outer facets of the root cells (“outer” here defined as facing the medium/soil; Takano et al. 2010). In contrast, BORON TRANSPORTER 1 and 2 (BOR1 and BOR2) are 2 known directional transporters that bring boron (in the form of borate anions) from the cytoplasm into the apoplast (Noguchi et al. 1997; Takano et al. 2002; Miwa et al. 2013). They are located to the inner facets of the root cells (“inner” here meaning facing the root's vasculature; Takano et al. 2010). NIP5;1, BOR1 and BOR2 accumulate at low boron conditions, and loss-of-function mutations in their genes reduce plant growth under low boron conditions (Takano et al. 2006; Miwa et al. 2013). BOR1 is predominantly involved in loading boron to the xylem, while BOR2 also facilitates pectin crosslinking within the cell wall by borate. The *bor1-3 bor2-1* double mutant exhibits significant reduction in boron concentration in roots grown under low boron conditions (Takano et al. 2002; Miwa et al. 2013). Previous modeling, which incorporated the expression and localization patterns of these channels and transporters within a longitudinal cross-section of the root tip, showed that the resultant fluxes create a boron gradient along the root meristem toward the tip region, confirmed by laser ablation inductively coupled plasma mass spectrometry (LA-ICP-MS) measurements (Shimotohno et al. 2015). It was hence postulated that localized boron accumulation at the very root tip would be critical for new cell wall formation in the stem cell niche and apical meristem, whereas in more proximal regions of the root boron channels and transporters predominantly function to bring boron into the xylem and to distribute it to the internal cell files. Xylem loading was further studied by focusing on a single row of root cells from the epidermis to the xylem and including channel and transporter regulation dynamics. This work has shown that such a system of BORs and NIPs can efficiently generate robust and stable boron throughput. Moreover, it was shown that boron throughput can be stable even under diverse and variable environmental conditions, but only if the channels and transporters respond sufficiently quickly to changes in the intracellular boron concentration (Sotta et al. 2017).

Among the 7 *BOR* genes in *Arabidopsis thaliana*, *BOR3* is closest to *BOR1* and *BOR2* (Nakagawa et al. 2007b). However, the role of BOR3 in boron transport and root growth has until now not been characterized. Is BOR3 simply adding to the robustness of the system, or is it functionally redundant? To answer this question, we started to investigate if and how BOR3 can contribute to root growth.

## Results

### BOR3 contributes to root growth under low boron conditions in the absence of BOR1 and BOR2

**Figure 1:**
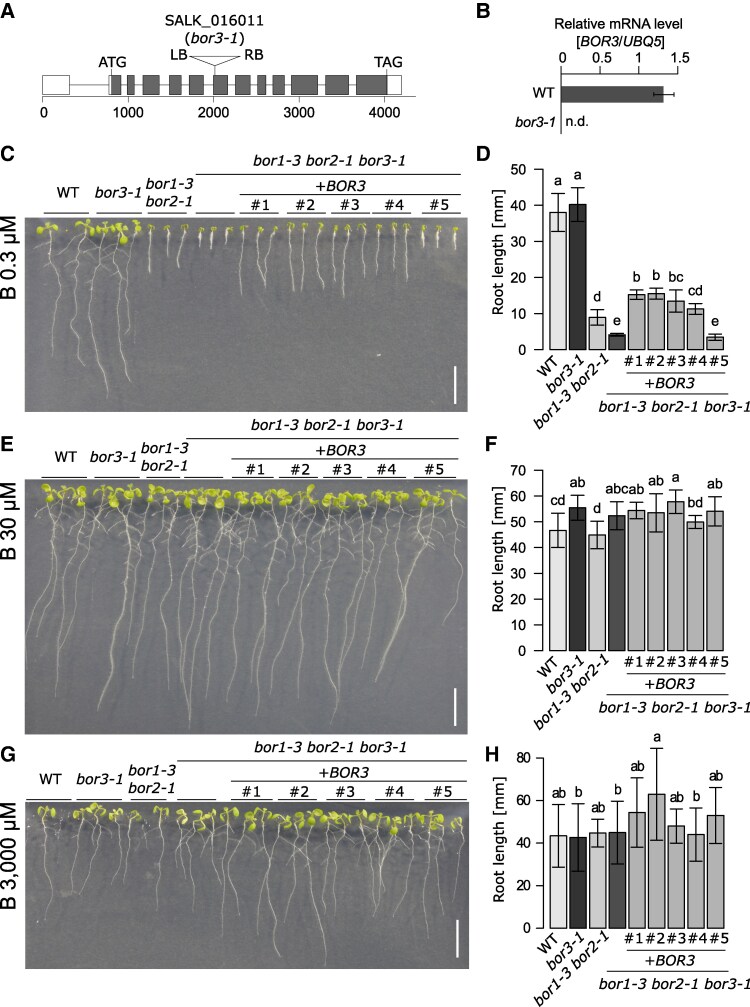
Effect of *bor3-1* mutation on growth. A) Schematic diagram of the *BOR3* gene structure and the T-DNA insertion site of *bor3-1*. B) RT-qPCR to detect *BOR3* mRNA accumulation in wild type and *bor3-1*. Total RNA was extracted from roots of 14-d-old seedlings grown under the 0.1 µM B condition. Values are mean ± SD for 3 biological replicates. C–H) Growth and root length measurements of *bor3-1* and the complementation lines. #1–#5 are independent transformant lines. Seedlings were grown for 7 d under 0.3 µM (C, D), 30 µM (E, F) or 3,000 µM (G, H) B conditions. Bars, 10 mm. Values are mean ± SD of 12 to 15 seedlings. There is no significant difference between groups sharing the same alphabet at *P* < 0.05 using Tukey–Kramer's test.

To unravel the role of BOR3 in the plant's physiology and growth, we first investigated the *bor3* mutant. We obtained the T-DNA insertion mutant SALK_016011 from the SALK T-DNA collection (Alonso et al. 2003) and established a T-DNA insertion homozygous line. Sequencing the T-DNA's flanking sequence revealed that the mutant line has a T-DNA insertion in the protein coding region on the sixth exon of BOR3 (Fig. 1A). From here on, we refer to SALK_016011 as *bor3-1*. When using RNA extracted from whole seedlings, we could detect, via RT-qPCR, *BOR3* transcripts in wild-type plants, but not in *bor3-1* mutant plants (Fig. 1B). We therefore consider *bor3-1* a *BOR3* knock-out mutant.

We first examined the contribution of BOR3 to plant growth under different boron conditions. Observation of seedlings 7 days after germination revealed that the *bor3-1* mutation does not have a detrimental effect on root elongation under either low (0.3 µM boric acid) (Fig. 1C and 1D), normal (30 µM boric acid) (Fig. 1E and 1F), or high boron conditions (3,000 µM boric acid) (Fig. 1G and 1H). This robustness can be explained by the presence of the transporters BOR1 and BOR2, which are known to significantly contribute to boron uptake and root growth under low boron conditions (Takano et al. 2002; Miwa et al. 2013). To further investigate the possible function of BOR3, we next examined the effect of BOR3 on root growth when both the other transporters are absent. Compared to the *bor1-3 bor2-1* double mutant, whose root elongation was limited to about 30% of the wild type under low boron conditions, the *bor1-3 bor2-1 bor3-1* triple mutant exhibited significantly poorer root elongation under low boron conditions (Fig. 1C and 1D). Introducing a genomic fragment of *BOR3* into *bor1-3 bor2-1 bor3-1* fully recovered the *bor1-3 bor2-1* root elongation under low boron conditions in 4 out of 5 independent transformants. In fact, in those 4 lines, root elongation was even enhanced when compared to the double mutant (Fig. 1C and 1D). These results indicate that although *BOR3* is not required for root elongation in wild-type plants under the controlled lab conditions shown here, its presence considerably promotes root growth under challenging low boron conditions when both *BOR1* and *BOR2* are absent.

### BOR3 does not present evident polarized localization

**Figure 2:**
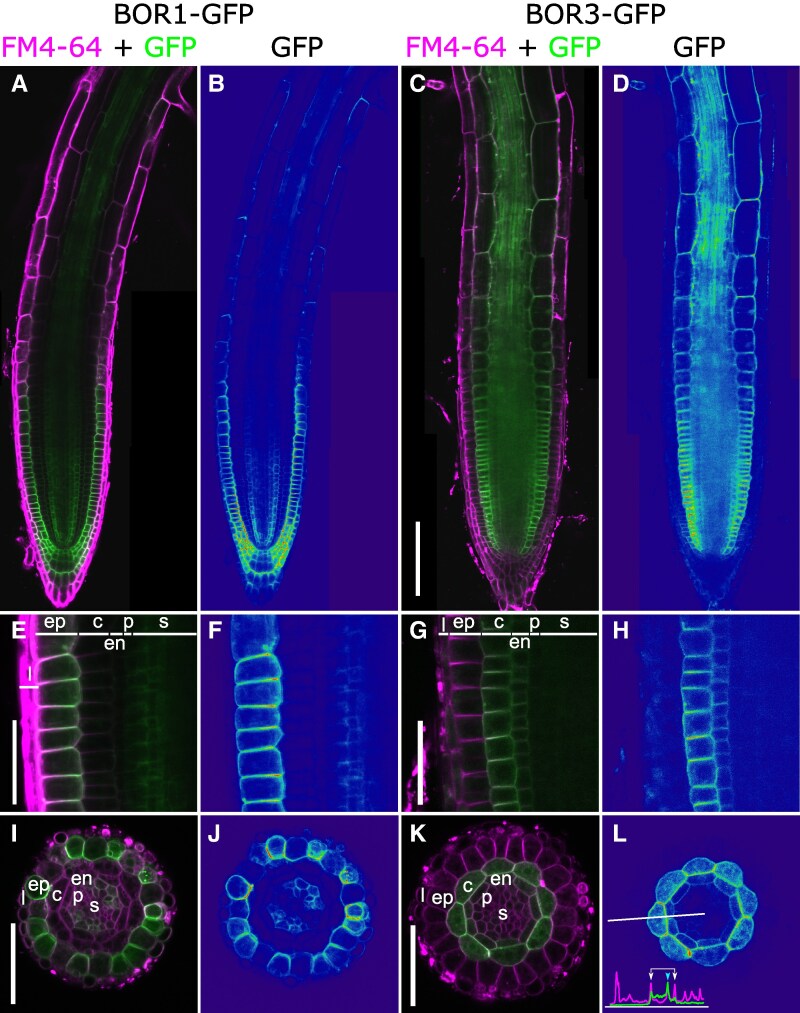
Expression of BOR3 in roots in comparison to BOR1 protein localization of BOR1-GFP (A, B, E, F, I, J) and BOR3-GFP (C, D, G, H, K, L) was observed by confocal microscopy. Seedlings were grown for 4 d under a 0.3 µM B condition. A–D) Root meristem up to elongation zone. In A–D, multiple high-magnification microscope images were tiled together to generate a composite image. E–H) Magnified images of root meristem cell layers. I–L) Cross-section within the root meristem zone. Inset in l represents the intensity profile of GFP (green) and FM4-64 (magenta) along the white line. White arrows represent the outer cortex and inner endodermis facets; the magenta arrow represents the inner cortex/outer endodermis facet. Bars: (A–D) 100 µm; (E–H) 30 µm; (I–L) 50 µm. Abbreviations: l, lateral root cap; ep, epidermis; c, cortex; en, endodermis; p, pericycle; s, stele.

The positive correlation between BOR3 and root growth under low boron conditions suggests that it is able to promote directed boron transport from the soil into the inner tissues, which is necessary for plant growth under such conditions. We would therefore expect BOR3 localization to be polar, predominantly located toward the inner cell facets, as is the case for BOR1 and BOR2 (Takano et al. 2006; Miwa et al. 2013). To characterize BOR3 localization patterns, we generated stable transformants which express BOR3-GFP driven by the putative *BOR3* promoter in the *bor3-1* background. We generated 3 independent transformant lines, all of which exhibited a consistent localization pattern. For the sake of comparison, we also observed BOR1-GFP (Fig. 2A, 2B, 2E, 2F, 2I, 2J). Under low boron conditions, BOR3-GFP fluorescence was observed in the cortex, endodermis, pericycle and stele (Fig. 2C, 2D, 2G, 2H). The fluorescence was not evident in the epidermis, quiescent center, columella, and lateral root cap. The most intense GFP fluorescence was observed in the cortical cells within the root meristem zone. As is the case for other already-reported *BOR* genes (*BOR1*, *2*, and *4*) (Miwa et al. 2007, 2013; Takano et al. 2010), observed GFP fluorescence is accumulated on the PM, supporting the notion that BOR3 is a plasma membrane protein.

Contrary to expectations, BOR3-GFP did not exhibit a clear polar localization (Fig. 2C, 2D, 2G, 2H), contrasting qualitatively to BOR1-GFP that exhibits evident inward polar localization (Fig. 2A, 2B, 2E, 2F) (Takano et al. 2010). From these observations, however, it is not possible to exclude that BOR3 localization could present a weak polarity (that is, being slightly more located on one side than the other) that went undetected in the longitudinal confocal view. Two factors render polarity difficult to assess quantitatively. First, inner and outer localization at neighboring cells can be difficult to distinguish. Second, fluorescence intensity drops when imaging deeper tissues, due to optical disturbance by tissues above the observation focus. These factors could contribute to masking actual polarity patterns. To address the first issue, we performed plasmolysis to physically separate the plasma membranes of neighboring cells (Figure S1). This process, however, triggered a loss of polarity in BOR1-GFP, which we used as a positive control for polar localization. This indicates that native polarity cannot be assessed under plasmolysis. The lack of polarity in BOR3, therefore, cannot be fairly interpreted under the plasmolysis condition. To address the second issue, we prepared physical cross-sections of root meristem regions and observed them using confocal microscopy. The cross-sections show specifically in the cortical cells a stronger BOR3-GFP fluorescence at the inner facet compared to the outer facet (Fig. 2K and 2L). In contrast, the endodermis and vasculature did not present such an inward polarity. It is however not possible to conclude from this observation that BOR3 has an inward polarity in the cortex but not in the endodermis, since the fluorescent signal on the outer facets of the endodermis is difficult to distinguish from the signal on the inner facets of the cortex; while the epidermis, which does not seem to express BOR3, is not likely to contribute any signal. Apparent polarity in the cortex could hence be due to incorrectly assigning part of the endodermal outer facets’ signal to the inner facets of the cortex. Because it is not possible using such confocal images to fully disentangle the contribution of each cell membrane to the signal at the interface, we more carefully analyzed the polarity in the following way: aggregate signals across cell interfaces (comprising both membranes) were quantified, which was then used to calculate a fluorescent intensity ratio, defined as (“outer cortex” + “inner endodermis”)/( “inner cortex & outer endodermis”) (Fig. 2L, inset). Under the limiting assumption of negligible accumulation in the epidermis and pericycle, this ratio is expected to be 1 if the localization of BOR3 is perfectly apolar in both the cortex and endodermis, independent of relative accumulation levels. We found an intensity ratio of 1.02 ± 0.18 (mean ± standard deviation [SD], *n* = 8), which does therefore not exclude the possibility that BOR3 is distributed apolarly in both cell types, with equal amounts of BOR3 localized at both the outer and inner facet. Although this analysis cannot provide a definitive answer, it highlights that the apparently stronger signal in the inner cortex compared to outer cortex cannot be straightforwardly interpreted as an inward localization of BOR3 in cortex. Based on these observations, we conclude that BOR3 likely presents no or very weak inward polarity.

### BOR3 expression does not present boron-dependent regulation

**Figure 3:**
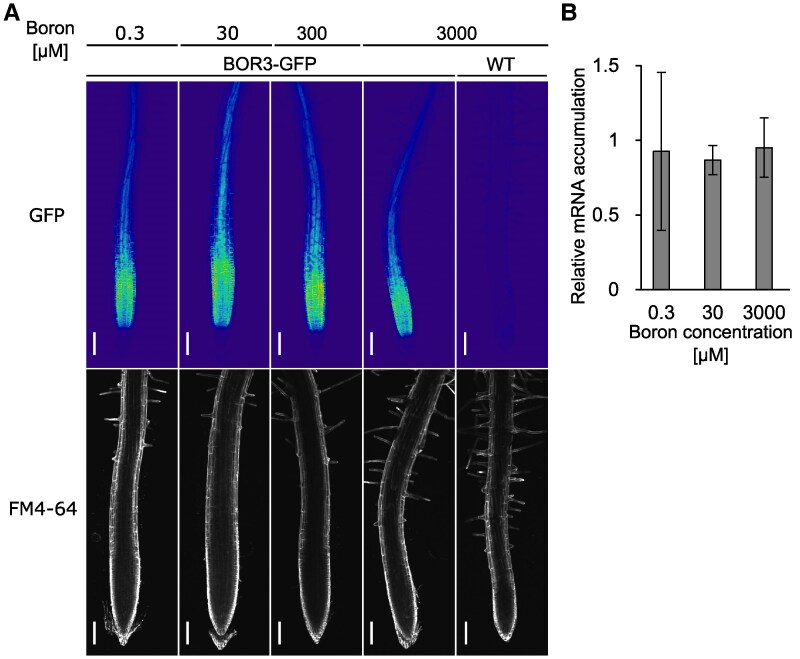
*BOR3* expression is not regulated by boron. A) Confocal images of BOR3-GFP fluorescence in primary roots of 6-d-old seedlings grown under various boron conditions. The wild-type root (WT) was included as a negative control. Plasma membrane was visualized with FM4-64 staining. Bars, 100 µm. B) RT-qPCR for *BOR3* mRNA accumulation. Total RNA was extracted from roots of 14-d-old seedlings grown under the indicated boron conditions. Values are mean ± SD for 3 biological replicates.

Because excess boron in planta causes cytotoxicity, expression of the other BORs with inward polarity, *BOR1* and *BOR2*, is downregulated through reduced translation and/or increased protein degradation in response to high boron environments (Takano et al. 2005; Miwa et al. 2013). This downregulation is regarded as a safeguard against taking up excess boron (Sotta et al. 2017; Aibara et al. 2018). GFP-tagged BOR1 and BOR2 can be observed to be localized on the PM under low boron conditions (0.1 to 0.3 µM B), whereas treatment with 100 µM B induces degradation of those proteins, causing them to become absent from the PM (Takano et al. 2005; Miwa et al. 2013). We analyzed BOR3-GFP in the primary roots grown under various boron conditions and compared the subcellular localization and fluorescent intensity. The fluorescent pattern of BOR3-GFP was not significantly affected by the specific boron conditions (Fig. 3A). The signal remained on the PM even under boron conditions as high as 3,000 µM B. For conditions varying from 0.3, 30, 300 to 3,000 µM B, no dose-dependent difference in GFP intensity were noticeable, indicating that *BOR3* expression is not boron-dependent. This was further corroborated through reverse transcription quantitative PCR (RT-qPCR) measurements that showed stable averages of relative mRNA accumulation at different boron concentrations (Fig. 3B).

### BOR3 is a borate exporter

**Figure 4:**
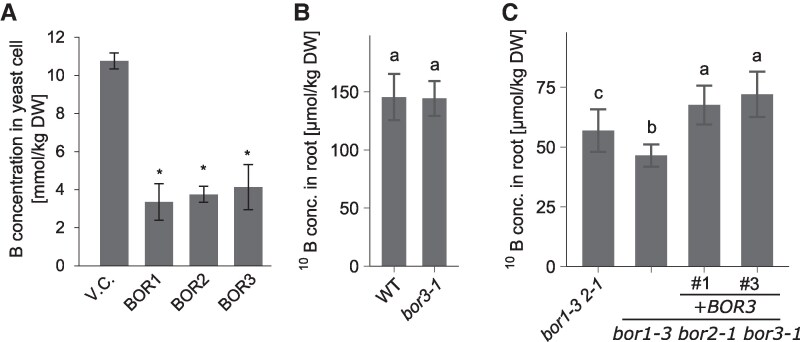
Boron transport activity of BOR3 (A) Boron concentrations in yeast cells expressing BOR3. Yeast strain *ΔScBOR1* was transformed with pYES2 (vector control) or pYES2 carrying BOR1, BOR2, or BOR3. Cells were incubated in SG medium with 1 mM boric acid for 60 min, after which the soluble boron concentration in the cells was determined by ICP-MS. Values are mean ± SD for the measurements from 3 independent transformants. Asterisks indicate significant difference from the vector control at *P* < 0.05 using Dunnett's test. B, C) Boron uptake assay with the stable isotope tracer ^10^B. Seedlings were grown for 15 d on medium with ^11^B and transferred to medium containing 0.3 μM ^10^B. 24 h after transfer, root ^10^B concentration was measured by ICP-MS. Values are the mean ± SD of the measurements from 6 to 18 biological replicates. B) No significant difference was detected using Welch's *t* test at *P* < 0.05. C) There is no significant difference between groups sharing the same alphabet using Tukey–Kramer's test at *P* < 0.05.

Despite *BOR3* not being boron-regulated, its beneficial role in the triple mutant background reveals that it does act as a boron transporter. To quantify the borate transporter activity of BOR3, we transformed ScBOR1 knock-out yeast strain 1169 (Winzeler et al. 1999; Takano et al. 2002) with a construct expressing BOR3 cDNA driven by the GAL1 promoter, to generate a yeast strain expressing BOR3. Cells at mid-log phase were incubated for 60 min in SG medium containing 1 mM boric acid, after which cellular soluble boron levels were measured using inductively coupled plasma mass spectrometry (ICP-MS). Yeast strains expressing BOR1 or BOR2 were included as positive controls. Cellular boron concentrations were significantly reduced in cells expressing BOR3, as well as those expressing BOR1 or BOR2, compared to the vector control (Fig. 4A). Boron was reduced up to 62% in these BOR3 expressing yeast cells, a comparable reduction that occurred due to BOR1 (69%) or BOR2 (65%), indicating that BOR3 displays borate export activity.

To investigate the contribution of BOR3 to boron uptake in planta, we measured root boron uptake of the mutants using the stable isotope tracer ^10^B. The boron uptake in *bor3-1* was not significantly different from that of the wild type (Fig. 4B). On the other hand, boron uptake of the *bor1-3 bor2-1 bor3-1* triple mutant was significantly lower than that of the *bor1-3 bor2-1* double mutant, while it was restored in the *bor1-3 bor2-1 bor3-1 BOR3* complementation line (Fig. 4C). These results indicate that BOR3 contributes to boron uptake in the absence of BOR1 and BOR2.

### Constraints on directional transport—the importance of being (a)polar

We have established BOR3's role as a directional boron transporter, exporting borate from the cytosol into the apoplast. It is difficult, however, to reconcile BOR3's positive contribution to root growth, which implies a role in directed boron transport through the root tissue, with its apparent negligible polar localization. In plant biology, it has long been taken as a ground truth that to establish a flux up a gradient over multiple cells, clear polar localization of directional transporters is a necessary requirement (Mitchison 1980). We first considered the possibility that altered BOR3 localization in *bor1-3 bor2-1* may contribute to boron uptake. However, the localization of BOR3-GFP in the *bor1-3 bor2-1 bor3-1* triple mutant background was indistinguishable from that of the wild type (Figure S2).

**Figure 5:**
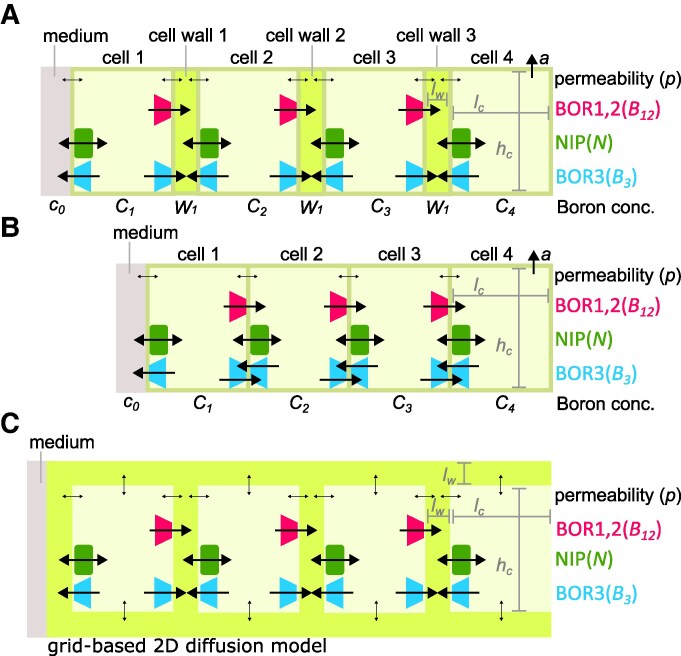
Schematic illustration of the mathematical models used to simulate boron transport in roots. Cross-sectional row of cells extending from the epidermis (left) to the xylem (right); schematics not to scale. Localization and polarity of boron channel and transporters in the wild type are shown. Arrows represent direction of active transport and diffusive transport. Corresponding transport parameters are indicated in parentheses. A, B) 1D ODE models, which consider transport between each neighboring compartments. Variables for the boron concentration in each compartment are shown at the bottom. A) Full model including all transporters as well as cell walls. B) An unrealistic model excluding cell walls to evaluate their role. C) Grid-based 2D partial differential equation model that includes anticlinal cell walls. Parameters provided in Table 1.

**Figure 6:**
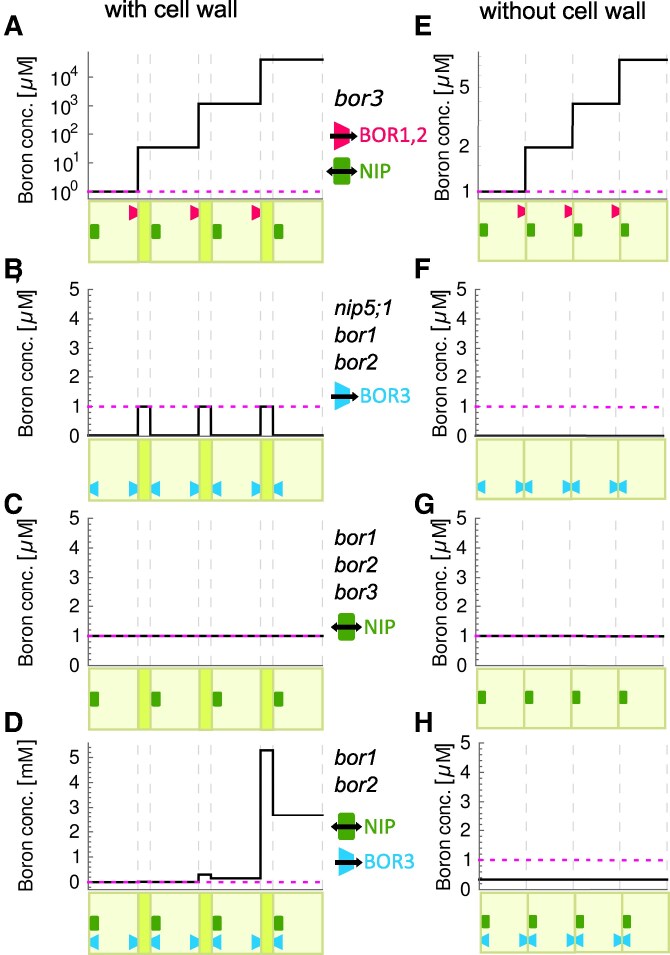
Building up gradients by synergy, and the essential role of the cell wall. A–H) Simplified quasi-1D models with (A–D) and without (E–H) cell wall. Steady-state for 4 different transporter scenarios: A, E) Channel polarly expressed to the outside (“NIP,” green) with polarly expressed polar transporter (“BOR1,2” magenta), yielding gradient; B, F) Apolar located polar transporter (“BOR3,” cyan) on itself does not generate a gradient in both cell wall scenarios; C, G) A passive channel alone is unable to generate gradients in both cell wall scenarios; D, H) combining “BOR3” with “NIP” gives rise to a gradient when the cell wall is present (D), but not otherwise (H).

To understand better what the basic constraints are for directed transport, we sought to explore the role of apolar efflux transporters mathematically and computationally. For this, we simplify the system to a quasi-2-dimensional (2D) strip of cells, portraying a transversal row of cells starting from the outer epidermal cell in contact with the medium, and extending up to the xylem loading cell (pericycle) (Fig. 5A). We queried, using such a simplified system, if the combination of exporters—either polar or apolar—together with the action of a bidirectional channel (NIP5;1) could or could not ensure boron uptake from the soil into the root tissue. As previously shown (Sotta et al. 2017), if such a strip of cells is endowed with outer-localized bidirectional channels (NIP5;1) and polar inner exporters, as BOR1 and BOR2 are known to be, this results in concentration increases within the tissue. That is, in such a scenario, cells positioned furthest away from the soil can present concentrations that are much higher than the concentration in the soil (Fig. 6A). For default permeability values (Table 1), such a system efficiently builds up a gradient, with the concentration in the fourth internal cells reaching 4 orders of magnitude higher concentrations than that of the medium (here, shown normalized to unit value).

**Table 1:** Model parameters.

Category	Parameter	Unit	Description	Default
Transporter	*N*	µm s^−1^	NIP5;1 activity	1
	$B_{12}$	µm s^−1^	BOR1 + BOR2 activity	1
	$B_{3}$	µm s^−1^	BOR3 activity	1
	$P_{B3}$	—	Polarity index for BOR3. 1 = perfectly inward, 0 = apolar, −1 = perfectly outward	0
Cell size	$l_{c}$	µm	Cell width	10
	$l_{w}$	µm	Cell wall width	0.5
	$h_{c}$	µm	Cell height	20
Other	*p*	µm s^−1^	Membrane background permeability of boron	0.03
	*a*	µm s^−1^	Xylem loading rate (in the last cell)	0.5
	$c_{0}$	µM	Boron concentration in medium	1

We then sequentially “deconstructed” this functional situation to pinpoint the necessary conditions for gradient establishment. First, we observe that if the system only expresses apolar exporters—an extreme interpretation of the BOR3 expression pattern—it would not be able to generate any gradient: cellular concentrations do not rise within the tissue, although some modest concentration accumulation is formed in the cell walls between cells in a nongraded fashion (Fig. 6B). Indeed, this result can be understood to be a consequence of the action of the exporters being equal on both sides of the cell, combined with the membrane permeability rates also being equal on all sides. As a consequence, the transporters do increase the flux of boron, but at the tissue scale this is done in a nondirected manner. Effectively, this localization pattern promotes diffusive behavior of the substrate in an undirected fashion. Likewise, a tissue lacking any exporters, endowed only with the bidirectional channels (NIP5;1) polarly located outwards, is again incapable of generating gradients (Fig. 6C). In this case, this is a consequence of the bidirectional channel, despite being polarly localized outward, providing only a diffusive contribution over that membrane itself—that is, it only augments boron permeability in an undirected fashion over one of the cell's facets. In short, it merely increases diffusive fluxes over certain interfaces, which cannot lead to the buildup of a gradient.

We next combine these 2 transport mechanisms, each of them being intrinsically diffusive (ie bidirectional channels and perfectly apolar exporters), which on their own cannot manifest gradient formation. Counterintuitively, a clear gradient arises (Fig. 6D). The maximum concentrations within this gradient are of comparable magnitude to the gradient formed in the “default” case, which included NIP5;1 and BOR1/2 (Fig. 6A). This tissue-level pattern emerges due to an iterative process of accumulated apoplastic boron being facilitated to diffuse into its neighboring, more internal cell, which, despite reaching lower concentration levels than its outer apoplastic cell wall, will then actively efflux boron to both sides via BOR3, resulting in a boron concentration increase in its inner cell wall. Through such a succession of iterations between cell–apoplast–neighboring cell, a gradient can arise. Following this reasoning, it becomes clear that the apoplastic compartment is vital to enable the synergistic action between the 2 “diffusive” transport systems during gradient formation. This insight can be verified though modeling, by repeating our analysis, but now using a model that lacks the apoplast, with cells being separated by plasma membrane only (Fig. 6E–6H). This artificial situation, which explicitly excludes the apoplastic compartment, although not capturing real plant tissue, allows us to analyze the specific contribution of the apoplastic space to the buildup of a gradient and directed boron transport. Indeed, the cell-wall-free model does not generate a gradient, instead presenting a flat profile (Fig. 6H), even though the same transporter localization and permeability values were used. It illustrates that by using models which do not take the apoplast into account, erroneous conclusions could be drawn that only a plant tissue with polarly localized directional transporters is capable of generating a gradient, and that an apolar localized exporter is insufficient (Fig. 6F), even if NIP5;1 is present (Fig. 6H).

### Coalition of diffusive processes at different spatial scales can generate a directed flow on a multicellular level due to the apoplast

**Figure 7:**
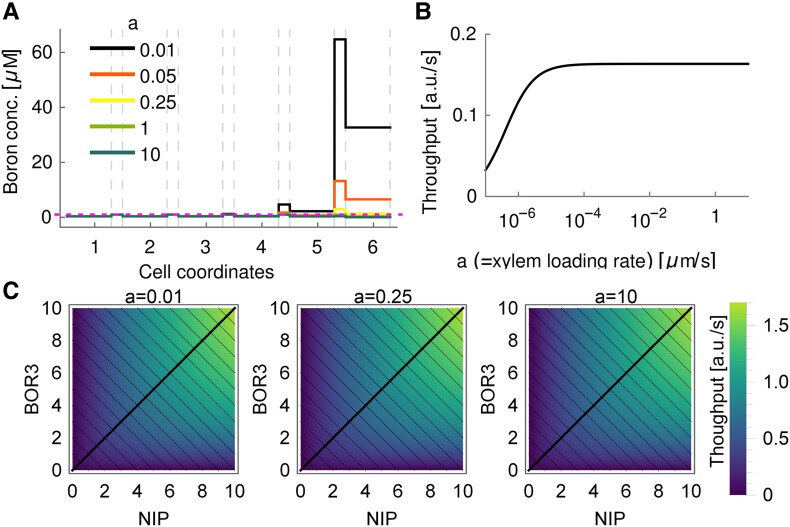
Contribution of BOR3 to boron uptake and the functionality of the components of the diffusive alliance transport. Relationship between the xylem loading rate and (A) the boron concentration in each compartment; and (B) the throughput. A) Boron concentration in each cell and cell wall compartment are shown for different xylem loading rates (in µm s^−1^). B) Throughput as a function of the xylem loading rate. C) Coalition of diffusive processes at different scales allowing for directed transport: Boron throughput through the cell row for all combinations of NIP (*x*-axis) and BOR3 (*y*-axis) levels (in µm s^−1^) for 3 different scenarios of xylem transport rates (A, shown above graphs). Main diagonal represents equal rates of NIP and BOR transport. Off diagonal lines represent conserved total transporter capacity, showing that given a certain capacity, the system only achieves significant net throughput through a combination of both diffusive transport processes.

In mature root tissues, boron transporters are responsible for loading the xylem with this essential mineral that will then be delivered to all growing tissues of the plant (Takano et al. 2002). The net throughput through the root system is a physiologically critical measurement, and possibly dependent on the convection within the xylem (Hopmans and Bristow 2002). Given that boron loading to the xylem was not considered in the model above, we next implemented this convection through an “effective xylem loading” term. This term removes boron from the top of our last, innermost cell, functionally merging the characteristics of the pericycle and xylem. We then asked if BOR3 activity on its own (considering it to be perfectly apolar) could contribute to boron throughput across such a tissue segment, and how this might depend on the xylem convection rate experienced in the last cellular compartment. By simulating different effective xylem loading rates spanning a large range of values (from 10 to 0.01 µm s^−1^), we first observe that the amounts of boron accumulated toward the proximal end of the gradient greatly vary with the loading rates (Fig. 7A). The larger the loading rate, the shallower the gradient. To determine how this affects actual boron uptake—of physiological relevance for the plant itself—we plotted the system's throughput as a function of the loading rate (Fig. 7B). Interestingly, although the gradient is highly sensitive to the loading rate, a very robust boron throughput can be established that only marginally depends on the xylem loading rates, as long as at least a very small amount of at least 10^−5^ µm s^−1^ is present (Fig. 7B). Moreover, whereas for large loading rates the apparent gradient formed disappears (Fig. 7A, green line, *a* = 10), this is not because polarized transport has ceased, given that throughput is still very high.

We next asked if this throughput homeostasis could be a result of our particular choice of BOR3 directional permeability and/or NIP5;1-dependent diffusive permeability rates (note that these values are estimated from the literature and empirical data; see Sotta et al. 2017). We therefore explored how throughput is affected when BOR3 and NIP permeabilities are varied for 3 different xylem convection scenarios (Fig. 7C). All 3 scenarios yield a similar dependency of throughput on transport parameters, clearly revealing that both BOR3 and NIP action need to be present concomitantly for an apolar efflux transporter system to function. Increasing either BOR3 or NIP action on its own, while maintaining the respective coalition partner constant, will lead to an increase in throughput. This increase, however, saturates at a level limited by the coalition partner. Therefore, if one of the transporters is not active enough, the other cannot compensate for that decreased function on its own. Furthermore, the manner in which this synergy acts is effectively independent of the xylem convection. This parameter robustness points to a universal mechanism of polar transport via the coalition of diffusive processes at different scales. We find it useful to term this characterized mechanism *diffusive alliance transport* (DAT), to differentiate it from classical polar transport mechanisms, such as the well-known *polar auxin transport* (PAT) (van Berkel et al. 2013).

### Efflux transporter localization analysis: apolar or polar and all that is between

To fully understand the actual role of BOR3 for the plant, and given our uncertainty about whether or not, and if so, to which extent BOR3 is polar, we initially adopted the worst-case scenario in which we considered, through a mathematical model, what the role of BOR3 could be if it indeed acted in a purely apolar manner. Doing so showed that it could account for our empirical findings on the beneficial effect of BOR3 for root growth and, on the physiological level, for boron uptake. However, BOR3's expression pattern, as was shown in Fig. 2, could also be interpreted as presenting some degree of polarity, that is, that BOR3 protein is localized with a higher density on one of the cell's facets than on the opposite side. Due to the inherent difficulties quantifying this polarity ratio from microscopy data—as distinguishing neighboring membranes is nontrivial and easily misinterpreted—we sought to explore in silico what the role of a generic export transporter is when considering the entire spectrum of possible polarity distributions.

**Figure 8:**
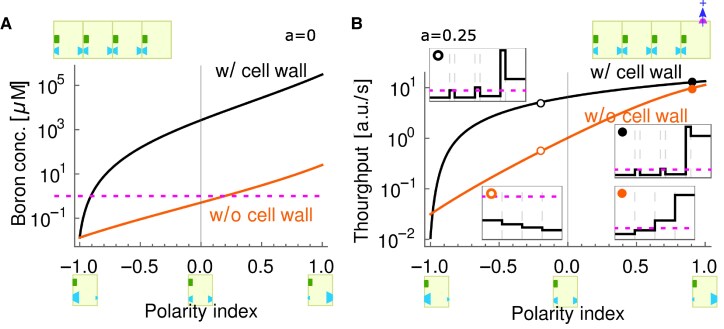
Generalized polarity model with and without cell wall. Relationship between BOR3 localization polarity and A) boron concentration in cell 4 with no xylem loading; and B) throughput for xylem loading rate *a* = 0.25. Insets in B) show boron concentrations in each compartment. Magenta lines represent boron concentration in medium (1 µM B).

We thus simulated the impact of a generic “BOR3” export transporter by varying it from outward localized (pure outward polarity, index −1) to completely apolar (both sides of the cell enriched evenly with the transporter, index 0), and even further, when polarity begins to invert to somewhat inward-orientated until pure inward polarity is reached (index +1). We assessed for any degree of polarity, ranging between −1 and 1, the resulting boron buildup, as measured by the boron concentration in the innermost cell in relation to the concentration in the medium, as well as by the boron throughput. We furthermore contrasted the results for the model with and without cell wall, to obtain a clearer understanding of the impact of the apoplast for the entire spectrum of polarized transporter localization. As expected from the model lacking cell wall (orange line, Fig. 8A), positive gradients can only be formed if the cells of the tissue present some degree of inward facing polarity. In this cell-wall-free scenario, when transporter localization is apolar, no gradient can form, and if the polarity inverts—even in minute terms—concentrations in the root will always be lower than the concentration in the medium. In stark contrast, the same system with the apoplast included presents both quantitatively and qualitatively different behavior: it maintains an efficient gradient when transporter localization is apolar (polarity level 0), with concentrations rising to quantitatively higher levels than those possible for the model without a cell wall endowed with inward-directed polarity. This reveals that, even when polarity is present, explicitly including the cell wall in a model will greatly increase resultant cellular concentrations. In accordance with our results above, we here again observe that at polarity index 0, corresponding to perfect apolarity, the system continues to maintain high relative internal concentrations. Surprisingly, this capacity does not disappear as the cells’ efflux transporter gradually becomes polarized outward. In fact, substantially increased relative boron concentrations are still present at an index value of −0.85, indicating that even when 85% of the exporter protein were to be localized on the outer side of the cells, the tissue—due to the apoplast and synergistic action between channels and efflux transporters—is still capable of establishing a positive gradient. This also translates into a highly robust uptake profile (Fig. 8B), showing that the model with the apoplast establishes efficient uptake over a wide window of polarities, whereas models without the cell wall consistently yield lower uptake capability for all polarity values. Moreover, the situation without cell wall presents a narrow range of functional polarities for which the system displays the capacity to elevate concentrations within the tissue to be above the level within the medium ($P_{B3}$ > 0.2 without cell wall vs. $P_{B3}$ > −0.9 with cell wall, Fig. 8B); and to establish effective throughput ($P_{B3}$ > 0.6 without cell wall vs. $P_{B3}$ > −0.2 with cell wall for a throughput of 5 a.u./s, Fig. 8B).

### Effect of anticlinal apoplastic connection on the directional transport

**Figure 9:**
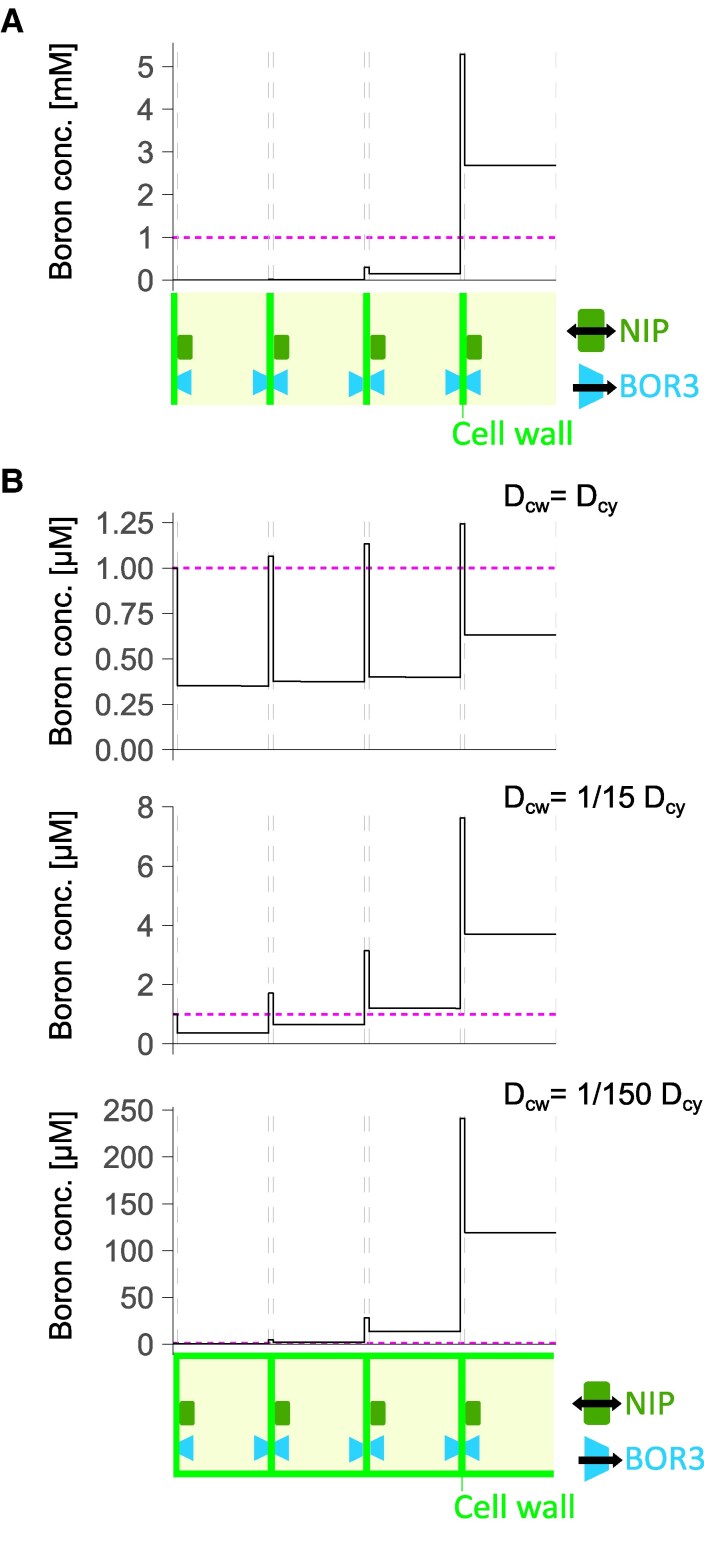
The effect of anticlinal apoplastic connections on boron transport driven by NIP5;1 and BOR3. Boron transport was simulated using a 2D PDE model. Steady-state boron distributions are shown for different tissue layouts. A) Model without anticlinal apoplastic connection, equivalent to the 1D ODE model. B) Model with anticlinal apoplastic connection. Plots show simulations results for 3 different cell walls to cytosol diffusion rate ratios. Magenta lines represent 1 μM, the boron concentration in the medium. Model details shown in Fig. 5C.

The insights above are derived from a simple 1-dimensional (1D) model in which cells and cell walls are linearly placed in a row spanning the outermost layer (epidermis) to the innermost layer (xylem-loading file). That is, cell walls are located between cells. In full 3-dimensional (3D) roots, the cells and cell walls are not merely ordered in rows but also stacked up in columns and in concentric radial rings. Simulating transport in such 3D structures is outside the scope of this study, as it would require extending our imaging to radial expression patterns and radial tissue asymmetries and accounting for complex 3D cell shapes. This would greatly increase the complexity of the model as well as the need for extra assumptions, thereby obfuscating our understanding of basic principles of uptake transport, for which a small 1D strip of cells is conceptually powerful. However, to generalize the validity of the basic behavior derived from our simplified 1D tissue, we acknowledge the potential of oversimplifications that could impact the transport, especially, in the context of how cells and cell walls are topologically connected. In the 1D model, boron can only move from the medium to the xylem by successive entries and exists into and from a cell into the neighboring periclinal cell wall and then into the next cell, and so forth. Crucially missing to this structure is the potential movement of boron via the contiguous anticlinal apoplast. To verify how this alternative path of movement might affect the diffusive alliance transport from NIP5;1 and BOR3, we carried out further simulations. We constructed a model that contains the same tissue structure as the previous 1D model but using a 2D grid instead of cells and periclinal cell walls being compartmentalized (Fig. 5C). We confirmed that this model behaves equivalently to the 1D model in terms of accumulating boron toward inside tissues (Fig. 9A). We next added the anticlinal layer of continuous cell wall above and below the cell row, which topologically changes the tissue, enabling anticlinal boron movement via the apoplast. This tissue context was used to simulate boron transport in the presence of NIP5;1 and BOR3 and compared to the previously obtained steady-state boron concentration distributions. Because diffusion rates can significantly differ between the cytoplasm and the apoplast (Kramer et al. 2007), we performed simulations for various boron diffusion rates in the cell wall. First, in the case where the diffusion rates in the cytosol (D_cy_) and cell wall (D_cw_) were set to be the same (an unrealistic assumption), the boron concentration in the cells became lower than in the medium, and no boron buildup was observed (Fig. 9B, top). We next simulated the case in which the diffusion in cell walls is lower than that of the cytoplasm. At a biologically more reasonable diffusion rate in cell walls (D_cw_ = 1/15 D_cy_, Kramer et al. 2007), we observed a buildup of boron concentration (Fig. 9B, middle). When the diffusion rate ratio was further reduced 10-fold (D_cw_ = 1/150 D_cy_), a more pronounced buildup of boron in the inner cells was observed (Fig. 9B, bottom). These results demonstrate that directional transport mediated by NIP5;1 and BOR3 is effective even in more realistic situations where the tissue is connected via anticlinal apoplast, and that the efficiency of this transport depends on the ratio of the diffusion coefficients between the cytoplasm and apoplast.

## Discussion

Our characterization of BOR3 was possible by combining empirical observations of genetic, developmental, and physiological properties with mathematical and computational analysis of transport mechanics. Genetics initially suggested a role of BOR3 in root growth promotion. We followed up on these observations and confirmed its transporter action as a borate exporter by measuring boron exclusion activity using yeast assays. But its functional role for the plant needed to be further addressed. From confocal imaging, BOR3 localization patterns seemed qualitatively distinct from the strikingly polar patterns documented for BOR1 and BOR2. This initially would suggest that it is not involved in boron uptake, which is, however, at odds with the mutant analysis. This led us to use mathematical reasoning to challenge the underlying dogma that export transporters rely on their polar localization to generate polar fluxes through plant tissue, with its polarity always assumed to be orientated into the direction of the transport. What we discovered—through this theoretical exploration—is that the coalition of apolar localized exporters (such as BOR3), with the presence of a bidirectional channel (the NIP5;1 outer expressed protein) *can yield physiologically relevant flows*.

The counterintuitive insight that 2 diffusive mechanisms operating at different scales—across a cell (BOR3) and across a membrane (NIP5;1)—are able to synergistically generate a qualitatively distinct tissue-level flux relies entirely on the existence of the apoplastic space. The implications of this are far-reaching: it is not necessary for transporters to display polarity in plant tissues for them to be candidates to drive an up-the-gradient flux, as long as they operate in combination with another diffusive process that is asymmetrically localized on cell membranes (a role played by NIP5;1 in this system). This mechanism that we term diffusive alliance transport (DAT) therefore provides a theoretical framework that allows us to reconcile the lack of any striking polarity in BOR3 localization with its role in contributing to root growth via boron transport.

Moreover, DAT also bears implications for animal tissues, as interstitial spaces would be able to act as the necessary intermediate compartments into which a mobile substrate could accumulate before entering neighboring cells (Zhou et al. 2012). Another important lesson, that this interdisciplinary study reveals, is that if we had employed a computational model lacking the apoplast to model transport within the plant, which is commonly done (Stoma et al. 2008; Bayer et al. 2009; Alim and Frey 2010), the conventional (and, in this case, erroneous) conclusion would have been drawn that only polarly localized directional transport is capable of generating a positive gradient away from the source and a flux up the gradient. If our boron transport modeling had not considered the cell wall, it would have led us to conclude that BOR3 needs to be polarly localized for it to be considered capable of contributing to boron uptake at all. Moreover, a model lacking apoplast would have erroneously concluded that nutrient uptake would only be substantial if the BOR3 inward polarity were striking (quantitatively high, see Fig. 8).

Despite this theoretical clarification of what is biophysically possible, the question remains whether BOR3 is functionally distinct from BOR1 and BOR2 in physiological terms. First, we consider its distinct polarity. We argue here that, despite our images showing it is not significantly polarly located, like BOR1 and BOR2, and despite it not “needing” to be polar to be functional (due to DAT), it is possible that the biological reality in regard to its actual polarity lies somewhere in between these extremes. Due to imaging limitations, we are not able to derive precise quantitative measurements. Instead, we used the model to analyze the effect that BOR3 would have if its polarity would vary between purely inward facing to purely outward-facing. BOR3 will likely have its localization somewhere in between purely inward facing and purely apolar. For all the values within this “plausible biological” regime, it should therefore be characterized as a functional transporter in plant tissue, even in the absence of BOR1 and BOR2. This reconciles the BOR3 localization pattern with our mutant analysis.

The theoretical analysis generated yet another unexpected insight. Even exporter localization that is enriched in the outward direction could generate—through DAT, that is the coalition with NIP5;1—a directed flow inwards. The implication is that if influx or diffusive permeability rates are asymmetric, then the direction *of the observed polarization of the exporter of a system does not immediately indicate the direction of the flow*. This illustrates how care must be given to “drawing arrows” across cells representing the visual polarity of an exporter enrichment and inferring only from this the direction of substrate movement, as is often done in research on polar auxin transport (PAT).

Our modeling insights were derived from simple in silico tissue structures. We explored 2 models, the first purely 1D, and the second, 2D. We have shown that taking into account a contiguous apoplastic space that extends from the medium inward still yields the same qualitative results as the simpler model. Note, however, that an unobstructed transport route will work against buildup of gradients and fluxes, as it can generate a backflow. It will therefore be interesting to consider in the future a full 3D tissue that also incorporates radial cell layers, as well as the cell rows and columns. In such a more detailed scenario, the angular polarities of transporters may also become relevant, as well as the break-of-symmetry within the vascular tissue that is characteristic for every plant species. Such an integrated model will not only require large-scale simulations that still include cell walls but also will need to be informed by sophisticated image analysis of 3D plant tissue data.

Taking together all our observations, we can conclude that BOR3 functions as an exporter of boron, although it is not boron responsive. The apparent redundancy of BOR3 with BOR1 and BOR2 can be lifted when we realize that its more “smeared” out polarity pattern—while still able to generate a directed flux—helps keeping higher boron levels in the cell wall, while lowering them in the cytosol itself. This could be relevant if we consider that boron is consumed in the cell wall, although the site of boron incorporation into RG-II is currently unknown (Miwa et al. 2013). Lowering the levels within the cells could also be relevant to ensure toxic cytoplasmic levels are not reached (Reid and Fitzpatrick 2009). BOR3 therefore not only contributes to boron flux robustness but could also act to fine-tune distributions on the subcellular and cellular scales with possible cell biological implications.

## Materials and methods

### Plant materials

The Col-0 ecotype was used both as the wild type and as the background for all mutant lines used in this study. *bor3-1* (SALK_016011) was obtained from the SALK institute (Alonso et al. 2003). T-DNA insertion homozygous lines were selected from the provided T2 population by PCR with the primer pairs BOR3 genomic ORF 5′ + BOR3 genomic ORF 3′ and BOR3 genomic ORF 3′ + pROK2_LBb1. For genotyping, the primer set BOR3_1230(732)_F + BOR3_1594(1006)_R was also used. For determination of the T-DNA insertion site, flanking sequences were determined by sequencing DNA fragments obtained by PCR with the primer pairs BOR3 genomic ORF 5′ + pROK2_LBb1 and BOR3 genomic ORF 5′ + pROK2_LBb1.

The mutant lines *bor1-3*, *bor2-1* and *bor1-3 bor2-1* are described in Kasai et al. (2011). The triple mutant line *bor1-3 bor2-1 bor3-1* was obtained by crossing *bor3-1* with *bor1-3 bor2-1*.

For the complementation test, the *bor1-3 bor2-1 bor3-1* triple mutant was transformed with a pKM19 plasmid that carries a genomic fragment containing the full length *BOR3* sequence, using the agrobacterium floral dip method (Clough and Bent 1998). The pKM19 plasmid was constructed as follows: PCR was performed using BAC F24P17 as a template with the primer set BOR3_genomic_F + BOR3_genomic_R to amplify a 6 kb genomic sequence containing the *BOR3* putative promoter, corresponding to 3,170 bp from the start codon, the ORF, and the 3′ UTR. The amplified fragment was cloned into pENTR/D-TOPO with pENTR Directional TOPO cloning kits (Invitrogen, USA), resulting in pKM18. The insert was subcloned into pMDC99 (Curtis and Grossniklaus 2003) by LR reaction, using the Gateway LR Clonase II Enzyme mix (Invitrogen) and following the manufacturer’s manual, resulting in pKM19 for plant transformation. Similarly, for BOR3-GFP lines a genomic fragment from 3,172 bp upstream of the *BOR3* ORF until the end of the ORF but without the stop codon was cloned into pENTR/D-TOPO, using the primer set BOR3_genomic_F2 + BOR3_genomic_R2, and subcloned into pGWB504 (Nakagawa et al. 2007a) by LR reaction, resulting in pST5. All primers used are listed in Table S1. After plant transformation, homozygous lines with single T-DNA insertion were selected based on segregation ratios of hygromycin B resistance. The T4 generation was used for the experiments.

### Growth conditions

For culture with solid medium plates, MGRL medium (Fujiwara et al. 1992) was supplemented with 1% sucrose (Wako, Osaka, Japan) and solidified with 1.5% gellan gum (Wako). The boric acid concentration was modified depending on the individual experimental designs. Seeds were surface-sterilized with 70% ethanol for 5 min, and then with 95% ethanol for 1 min, and were sown on the plate medium. After a 4 °C treatment for 2 d the plates were vertically placed into incubators set to 22 °C and a 16 h light/8 h dark cycle.

### Observation of protein localization

Fluorescent images were captured with confocal microscopes (FV1000, Olympus; and SP8 TCS, Leica). Plasma membrane was stained with 4 µM FM4-64 (Invitrogen) in PBS buffer and observed with 559 nm excitation and 584 to 684 nm emission. Cell wall was stained with 10 μg/mL propidium iodide aqueous solution for 3 min and observed with 559 nm excitation and 575 to 675 nm emission. GFP fluorescence was observed with 473 nm excitation and 485 to 545 nm emission. Root cross-sections were prepared and observed as described in Sotta et al. (2017).

For plasmolysis experiments, roots were soaked in 0.8 M mannitol solution for 1.5 h, and cell walls were stained with 0.1% calcofluor white in 0.8 M mannitol solution for 0.5 h. GFP was observed with 488 nm excitation and 500 to 530 nm emission, and calcofluor white was observed with 405 nm excitation and 430 to 470 nm emission.

### Boron transport activity assay with yeast

*Saccharomyces cerevisiae ΔScBOR1* strain 1169 (Winzeler et al. 1999; Takano et al. 2002) was transformed with pTF522, which expresses BOR3 cDNA driven by the GAL1 promoter. To generate pTF522 (BOR3 CDS in pYES2), *BOR3* CDS in cDNA was amplified with the primer set BOR3_CDS_KpnI_F + BOR3_CDS_SphI_R. The PCR products were digested with *Kpn*I and *Sph*I and inserted into pYES2 vector (Invitrogen). Transformation of yeast and measurement of soluble boron concentration by ICP-MS are described in Miwa et al. (2013).

### Boron uptake assay using isotope tracer

Arabidopsis seeds were sown on medium plates with embedded nylon mesh for rapid transfer, as described in Yoshimoto et al. (2016). Seedlings precultured on MGRL medium plates containing 30 µM ^11^B(OH)_3_ for 14 d were transferred to MGRL hydroponic culture solution containing 0.3 µM ^11^B(OH)_3_. After 24 h, the hydroponic solution was replaced with MGRL solution containing 0.3 µM ^10^B(OH)_3_. After 24 h, whole roots were sampled after brief rinse with ultrapure water. Samples were dried in an oven at 65 °C for more than 24 h, and the dry weight was measured and recorded. Samples were digested with HNO_3_ (density 1.38, for B determination; Wako, Osaka, Japan) with addition of H_2_O_2_ at 110 °C. The resulting pellets were dissolved in 5 mL of 0.08 N HNO_3_ and subjected to ICP-MS (Agilent 7800). The ^10^B content per root dry weight was calculated to evaluate the 24-h boron uptake.

### Mathematical modeling

#### Boron transport within a 1-dimensional model (row of cells)

This model consists of a set of coupled ordinary differential equations (ODEs) that describe the dynamics of the intracellular and apoplastic boron concentration within a single row of cells along a cross-section of the root. This cross-sectional row starts at the epidermis in contact with the medium (the outer side) and extends up to the inner tissues, terminating at the xylem (the inner side) (Fig. 5A). Cytoplasmic boron concentrations $C_{i}$ in each cell *i* and apoplastic boron concentrations $W_{i}$ in the adjacent inner cell wall of cell *i* are expressed as continuous variables for each well-mixed compartment. The model was developed using a similar modeling strategy as described in Sotta et al. (2017), with extensions to capture the additional transporter BOR3 ($B_{3}$). We define the extent of $B_{3}$ localization being polar or apolar through the polarity parameter $P_{B3}$


(1) $$P_{B3}=\frac{\mathrm{inwards}\;\mathrm{localization}-\mathrm{outwards}\;\mathrm{localization}}{\mathrm{inwards}\;\mathrm{localization}+\mathrm{outwards}\;\mathrm{localization}}\;.$$

A value of zero thus indicates perfectly apolar localization; *a* value of 1 captures localization that is fully confined to the inward facets; a value of −1 confinement to the outward facets; and values in between capture mixed polarity levels. The equations governing the time evolution of the boron concentrations as a function of transporter and channel levels and polarity then become:


(2a ) $$\frac{dC_{1}}{dt}=(\left(\;p\;+\;N)(c_{0}-C_{1})-(B_{12}+2B_{3})C_{1}+p(W_{1}-C_{1}\right))\frac{1}{l_{c}}$$

(2b ) $$\frac{dC_{i}}{dt}=(\left(\;p\;+\;N)(W_{i-1}-C_{i})-(B_{12}+2B_{3})C_{i}+p(W_{i}-C_{i}\right))\frac{1}{l_{c}}$$

(2c ) $$\frac{dC_{n}}{dt}=((\;p\;+\;N)(W_{n-1}-C_{n})-B_{3}(1-P_{B3})C_{n})\frac{1}{l_{c}}-aC_{n}\frac{1}{h_{c}}$$

(2d ) $$\begin{array}{r} \frac{dW_{i}}{dt}=((\;p(C_{i}-W_{i})+(\;p+N)(C_{i+1}-W_{i})+B_{12}C_{i}\\ +B_{3}(1+P_{B3})C_{i}+B_{3}(1-P_{B3})C_{i+1})\frac{1}{l_{w}} \end{array}$$

where *N*, $B_{12}$, and $B_{3}$ represent boron permeabilities due to *NIP5;1*, *BOR1/BOR2*, and *BOR3*, respectively. Cell 1 (with boron concentration $C_{1}$) is the outermost cell, in contact with the external medium/outermost cell wall that is kept at a fixed boron concentration $c_{0}$. Cell *n* ($C_{n}$) is the innermost cell that presents no further inwards transport or leakage, but unlike the other cells it is bestowed with an upward flux that could either be attributed to membrane transport or could be convective in nature. $C_{i}$ in Eq. 2 represents the boron concentration in all other cells (i=2,…,n−1). $W_{i}$ represents all cell walls flanking the cells on the inner side (i=1,…,n−1). Together this gives rise to in total 2n−1 coupled ODEs. All parameters are described and values given in Table 1. In this model, the boron concentration in a certain compartment is determined by the influx and efflux from and to their neighboring compartments. Models with different channel and transporter compositions were expressed by setting channel/transporter activity of absent channels or transporters to 0. Steady-state boron concentrations were calculated by solving $\frac{dC_{i}}{dt}=0$ for i=1,…,n and $\frac{dW_{i}}{dt}=0$ for i=1,…,n−1.

To understand the contribution of the cell walls to the dynamics, we purposefully analyzed the model behavior under the unrealistic simplifying assumption of an absence of cell walls (Fig. 5B). The following set of coupled ODEs were used to capture this cell-wall-free condition, using the same model structure, but now having boron flux directly occurring between neighboring cells:


(3a ) $$\frac{dC_{1}}{dt}=(\left(\;p+N)(c_{0}-C_{1})-(B_{12}+2B_{3})C_{1}+B_{3}(1-P_{B3})C_{2}+p(C_{2}-C_{1}\right))\frac{1}{l_{c}}$$

(3b ) $$\begin{array}{r} \frac{dC_{i}}{dt}=((\;p+N)(C_{i-1}-C_{i})-(B_{12}+2B_{3})C_{i}+B_{12}C_{i-1}\\ +B_{3}(1+P_{B3})C_{i-1}+B_{3}(1-P_{B3})C_{i+1}+p(C_{i+1}-C_{i}))\frac{1}{l_{c}} \end{array}$$

(3c ) $$\begin{array}{r} \frac{dC_{n}}{dt}=((\;p\;+\;N)(C_{n-1}-C_{n})-B_{3}(1-P_{B3})C_{n}\\ +B_{3}(1+P_{B3})C_{n-1}+B_{12}C_{n-1})\frac{1}{l_{c}}-aC_{n}\frac{1}{h_{c}} \end{array}$$

All numerical calculations were computed with Mathematica 11.3.

#### Boron transport within a 2-dimensional spatial model

To evaluate the impact of unobstructed boron diffusion within the anticlinal cell wall, we simulated boron transport using a grid-based 2D spatial model that also incorporates the flanking cell walls (Fig. 1C). These simulations resolve partial differential equations (PDEs) of free boron diffusion in the cell wall and cytoplasm as well as directed and permissive transport across the cell membranes due to transporters, channels, and PM properties. The simulation framework and parameters have been consolidated in a previous study (Shimotohno et al. 2015). The PDE model represents a geometric structure analogous to the 1D compartmentalized model, except for the addition of the anticlinal cell wall (Fig. 5C). Note that due to this continuous cell wall structure, a compartment-based ODE modeling strategy cannot be utilized. Simulations were run to obtain steady-state solutions. Wrapped boundary conditions are used in the vertical dimension to simulate the row as if it were within a continuous root tissue. To allow for direct comparison between the 1D and 2D models, we plot boron concentrations along a straight line crossing the center of the in silico cell row.

### Accession numbers

Sequence data from this article can be found in the GenBank/EMBL data libraries under accession numbers AT2G47160 (*BOR1*), AT3G62270 (*BOR2*), At3g06450 (*BOR3*), and AT4G10380 (*NIP5;1*).

## Supplementary Material

kiag463_Supplementary_Data

## Data Availability

The data that support the findings of this study are available from the corresponding author upon reasonable request.
