# Supplementary material for: Characterization and modeling of the borate transporter BOR3 suggest a mode of directional transport
Source: Plant Physiol. 2026 Jul 30;201(3):kiag463. doi: 10.1093/plphys/kiag463 (PMC13420508; doi:10.1093/plphys/kiag463)
Supplement: kiag463_Supplementary_Data [file kiag463_supplementary_data.pdf]

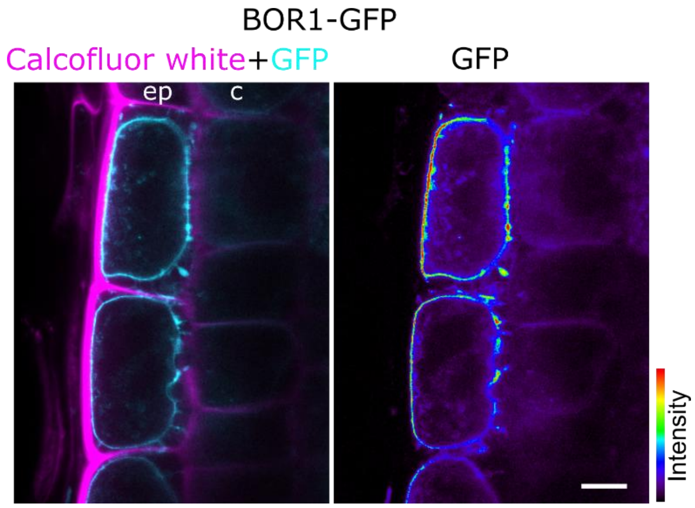

Supplementary Figure S1. Localization of BOR1-GFP in roots under plasmolysis
Protein localization of BOR1-GFP in roots under plasmolysis was observed by confocal microscopy. Seedlings were grown for 5 days under a 0.3 μM B condition. Plasmolysis was induced by 0.8 M mannitol treatment for 2 h. ep, epidermis; c, cortex. Bar: 10 μm.

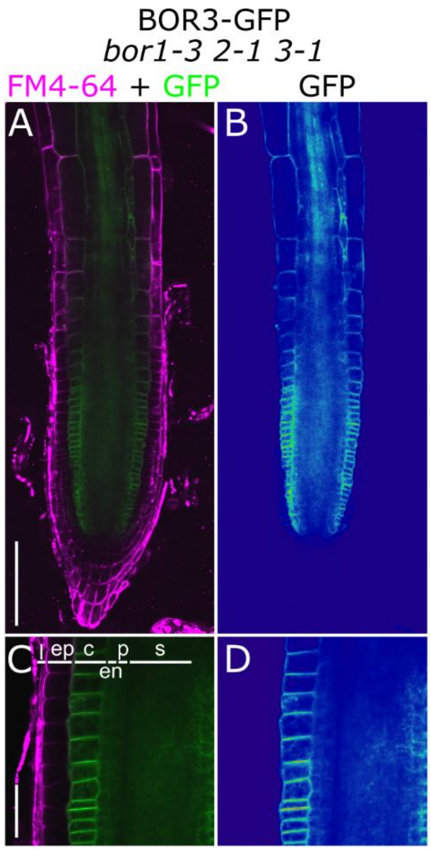

Supplementary Figure S2. Localization of BOR3 in *bor1-3 2-1 3-1* roots Protein localization of BOR3-GFP in *bor1-3 2-1 3-1* triple mutant was observed by confocal microscopy. Seedlings were grown for 5 days under a 0.3 µM B condition. (A–B) Root meristem to elongation zone. (C–D) Magnified images of root meristem cell layers. Bars: (A–B) 100 µm; (C–D) 30 µm. l, lateral root cap; ep, epidermis; c, cortex; en, endodermis; p, pericycle; s, stele.

Supplementary Table S1. Primers used in this study

Name	Sequence
**T-DNA insertion detection**	
**pROK2_LBb1**	GATGGCCCACTACGTGAACCAT
**pROK2_RB**	TAGTGACCTTAGGCGACTTTT
**BOR3_1230(732)_F**	AATGGTGGTAGTGTGGACT
**BOR3_1594(1006)_R**	AATGGTAAGCAGGAGGCTTTC
**BOR3 genomic ORF 5'**	GAGTGTTGTTTTGTAGGACACTGAAA
**BOR3 genomic ORF 3'**	TTCTTAAATCTCATAATGACAGGCAAAT
**Construction**	
**BOR3_genomic_F**	CACCCTGTCTAGAGGCTTACCAATTTGAATAA
**BOR3_genomic_R**	TGTACTCTTGGACTACTAAC
**BOR3_genomic_F2**	CACCTCAGAGGCTTACCAATTTGA
**BOR3_genomic_R2**	AACAGACTTTCCTAAATTAGAAAG
**BOR3_CDS_KpnI_F**	TGAGAGGTACCAGAGCC ATGGACGAAGCAGAGAGCTTTGTC
**BOR3_CDS_SphI_R**	GTTTTAGCATGCCTAAACAGACTTTCCTAAATTAGAAAGTCTC
**qRT-PCR**	
**Actin8_RT_F**	GCCAGATCTTCATCGTCGTG
**Actin8_RT_R**	TCTCCAGCGAATCCAACCTT
**UBC_RT_F**	CTGCGACTCAGGGAATCTTCTAA
**UBC_RT_R**	TTGTGCCATTGAATTGAACCC
**SAND_RT_F**	AACTCTATGCAGCATTTGATCCACT
**SAND_RT_R**	TGATTGCATATCTTTATCGCCATC
**BOR3rtpcr-F**	TTCAATCTCAAACCGGAAGG
**BOR3rtpcr-R**	TACCGTCGATCCACTTCCTC
